# Bis(2-amino-6-methyl­pyridinium) tris­(pyridine-2,6-dicarboxyl­ato)zirconate(IV) dihydrate

**DOI:** 10.1107/S1600536811003072

**Published:** 2011-02-02

**Authors:** Hoda Pasdar, Ali Ebdam, Hossein Aghabozorg, Behrouz Notash

**Affiliations:** a Department of Chemistry, Islamic Azad University, North Tehran Branch, Tehran, Iran; b Department of Chemistry, Shahid Beheshti University, G. C., Evin, Tehran 1983963113, Iran

## Abstract

In the title compound, (C_6_H_9_N_2_)_2_[Zr(C_7_H_3_NO_4_)_3_]·2H_2_O, the Zr^IV^ atom is nine-coordinated by three pyridine-2,6-dicarboxyl­ate ligands in a distorted tricapped trigonal–prismatic ZrN_3_O_6_ environment. The crystal packing is stabilized by inter­molecular N—H⋯O and O—H⋯O hydrogen bonds.

## Related literature

For background to proton-transfer compounds, see: Aghabozorg *et al.* (2008 ▶). For related structures, see: Aghabozorg *et al.* (2005 ▶); Daneshvar *et al.* (2008 ▶); Willey *et al.* (1998 ▶); Pasdar *et al.* (2010 ▶, 2011 ▶).
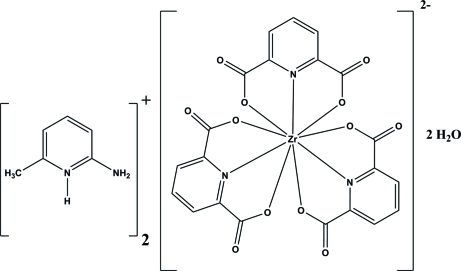


## Experimental

### 

#### Crystal data

(C_6_H_9_N_2_)_2_[Zr(C_7_H_3_NO_4_)_3_]·2H_2_O
*M* *_r_* = 840.87
Monoclinic, 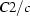

*a* = 18.719 (4) Å
*b* = 10.536 (2) Å
*c* = 18.781 (4) Å
β = 108.58 (3)°
*V* = 3511.0 (14) Å^3^
*Z* = 4
Mo *K*α radiation
μ = 0.39 mm^−1^
*T* = 298 K
0.35 × 0.30 × 0.25 mm

#### Data collection

Stoe IPDS II diffractometer
Absorption correction: numerical [shape of crystal determined optically (*X-SHAPE* and *X-RED32*; Stoe & Cie, 2005 ▶)] *T*_min_ = 0.870, *T*_max_ = 0.903
12273 measured reflections
4705 independent reflections
4284 reflections with *I* > 2σ(*I*)
*R*_int_ = 0.025

#### Refinement

*R*[*F* ^2^ > 2σ(*F* ^2^)] = 0.023
*wR*(*F* ^2^) = 0.060
*S* = 1.03
4705 reflections
271 parameters
H atoms treated by a mixture of independent and constrained refinement
Δρ_max_ = 0.33 e Å^−3^
Δρ_min_ = −0.27 e Å^−3^

### 

Data collection: *X-AREA* (Stoe & Cie, 2005 ▶); cell refinement: *X-AREA*; data reduction: *X-AREA*; program(s) used to solve structure: *SHELXS97* (Sheldrick, 2008 ▶); program(s) used to refine structure: *SHELXL97* (Sheldrick, 2008 ▶); molecular graphics: *ORTEP-3 for Windows* (Farrugia, 1997 ▶); software used to prepare material for publication: *WinGX* (Farrugia, 1999 ▶).

## Supplementary Material

Crystal structure: contains datablocks I, global. DOI: 10.1107/S1600536811003072/bt5467sup1.cif

Structure factors: contains datablocks I. DOI: 10.1107/S1600536811003072/bt5467Isup2.hkl

Additional supplementary materials:  crystallographic information; 3D view; checkCIF report

## Figures and Tables

**Table 1:** Hydrogen-bond geometry (Å, °)

*D*—H⋯*A*	*D*—H	H⋯*A*	*D*⋯*A*	*D*—H⋯*A*
O7—H7*B*⋯O3	0.87 (4)	2.09 (4)	2.938 (2)	164 (3)
O7—H7*A*⋯O6^i^	0.82 (3)	2.14 (3)	2.9568 (19)	173 (3)
N4—H4*B*⋯O1^ii^	0.86 (2)	2.57 (2)	3.1755 (18)	127.6 (17)
N4—H4*B*⋯O7	0.86 (2)	2.28 (2)	3.027 (3)	144.1 (18)
N4—H4*A*⋯O4^iii^	0.82 (2)	2.05 (2)	2.861 (2)	168 (2)
N3—H3*A*⋯O2^ii^	0.91 (2)	1.91 (2)	2.8194 (18)	175.1 (18)

Symmetry codes: (i) 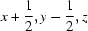; (ii) 
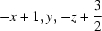
; (iii) 
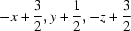
.

**Fig. 1.**
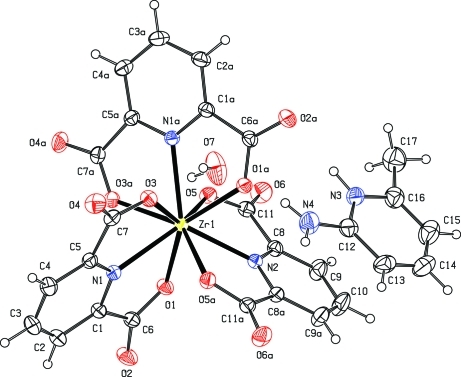
The molecular structure of (2a6mpH)2[Zr(2,6-pydc)3].2H2O with displacement ellipsoids drawn at 30% probability level.Symmetry code: (a) -x + 1, y, -z + 3/2.

**Fig. 2.**
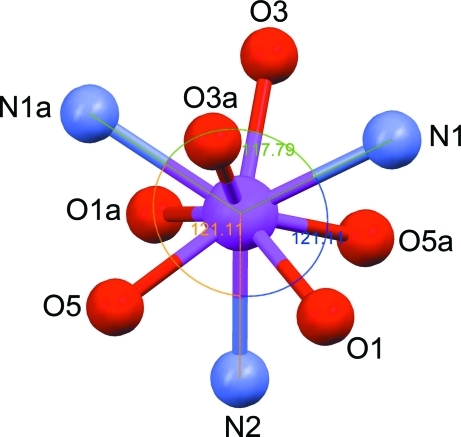
The coordination environment around ZrIV ion in the title compound. Symmetry code: (a) -x + 1, y, -z + 3/2.

**Fig. 3.**
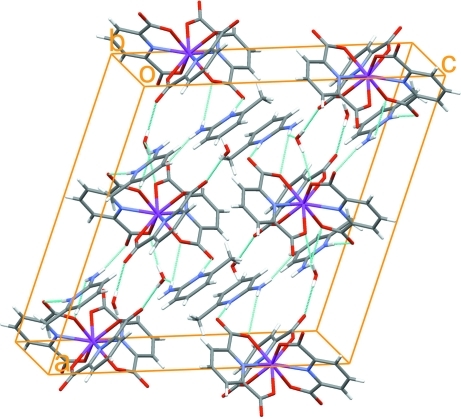
The packing diagram of (2a6mpH)2[Zr(2,6-pydc)3].2H2O. The intermolecular N—H···O, O—H···O hydrogen bonds are shown as blue dashed lines.

## supplementary crystallographic information

### Comment

Pyridine-2,6-dicarboxylic acid (pydcH_2_) was commonly used as an acid in proton transfer systems (Aghabozorg *et al.*, 2008). Continuing the path to synthesize proton transfer compounds, our group has focused on forming ion pairs between 2,6-pydcH_2_ and various organic bases (Pasdar *et al.*, 2010; Pasdar *et al.*, 2011). The structures of two proton transfer compounds containing [Zr^IV^(2,6-pydc)_3_]^2-^ moiety were reported with the counter cationic part of 2,6-pyridinediamine (Aghabozorg *et al.*, 2005) and 2,4,6-triamino-1,3,5-triazine (Daneshvar *et al.*, 2008), respectively. The structure of K_4_[Zr^IV^(2,6-pydc)_3_]_2_ has been reported by Willey *et al.* (1998).

We report herein the synthesis and crystal structure of (2a6mpH)_2_[Zr(2,6-pydc)_3_].2H_2_O by the reaction of ZrCl_4_, 2-amino-6-methylpyridine and 2,6-pyridinedicarboxylic acid in aqueous media. The molecular structure of the title compound is shown in Fig.1. The zirconium(IV) ion is coordinated by three pydc^2-^ ligands in a distorted tricapped trigonal prismatic geometry. The geometry around the zirconium(IV) centre in the title compound is shown in Fig. 2. The Zr—N and Zr—O bond lengths and angles are comprable with those previously reported (Aghabozorg *et al.*, 2005; Daneshvar *et al.*, 2008). The crystal packing diagram of (2a6mpH)_2_[Zr(2,6-pydc)_3_].2H_2_O is presented in Fig. 3. There are several intermolecular N—H···O, O—H···O hydrogen bonds which stabilize crystal structure of the compound (Table 1 and Fig. 3).

### Experimental

A solution of ZrCl_4_ (0.114 mg,0.5 mmol) in water (15 ml) was added to an aqueous solution of 2-amino-6-methylpyridine (0.114,1 mmol) and 2,6-pyridinedicarboxylic acid (0.504 mg, 3 mmol) in water (15 ml). Crystals of the title compound suitable for X-ray characterization were obtained after a few weeks at room temperature (m.p: 145 °C).

### Refinement

The hydrogen atoms bonded to N and O were found in difference Fourier map and refined isotropically without restraint. The C—H protons were positioned geometrically and refined as riding atoms with C—H = 0.93 Å and *U*iso(H) = 1.2 *U*eq(C) for aromatic C—H   and C—H = 0.96 Å and *U*iso(H) = 1.5 *Ueq*(C) for methyl groups.

### Crystal data

**Table:**

(C_6_H_9_N_2_)_2_[Zr(C_7_H_3_NO_4_)_3_]·2H_2_O	*F*(000) = 1720
*M**_r_* = 840.87	*D*_x_ = 1.591 Mg m^−^^3^
Monoclinic, *C*2/*c*	Mo *K*α radiation, λ = 0.71073 Å
Hall symbol: -C 2yc	Cell parameters from 4705 reflections
*a* = 18.719 (4) Å	θ = 2.3–29.2°
*b* = 10.536 (2) Å	µ = 0.39 mm^−^^1^
*c* = 18.781 (4) Å	*T* = 298 K
β = 108.58 (3)°	Prism, colorless
*V* = 3511.0 (14) Å^3^	0.35 × 0.3 × 0.25 mm
*Z* = 4	

### Data collection

**Table:**

Stoe IPDS II diffractometer	4705 independent reflections
Radiation source: fine-focus sealed tube	4284 reflections with *I* > 2σ(*I*)
graphite	*R*_int_ = 0.025
Detector resolution: 0.15 mm pixels mm^-1^	θ_max_ = 29.2°, θ_min_ = 2.3°
rotation method scans	*h* = −25→25
Absorption correction: numerical [shape of crystal determined optically (*X-SHAPE* and *X-RED32*; Stoe& Cie, 2005)]	*k* = −12→14
*T*_min_ = 0.870, *T*_max_ = 0.903	*l* = −25→25
12273 measured reflections	

### Refinement

**Table:**

Refinement on *F*^2^	Primary atom site location: structure-invariant direct methods
Least-squares matrix: full	Secondary atom site location: difference Fourier map
*R*[*F*^2^ > 2σ(*F*^2^)] = 0.023	Hydrogen site location: inferred from neighbouring sites
*wR*(*F*^2^) = 0.060	H atoms treated by a mixture of independent and constrained refinement
*S* = 1.03	*w* = 1/[σ^2^(*F*_o_^2^) + (0.0339*P*)^2^ + 1.0948*P*] where *P* = (*F*_o_^2^ + 2*F*_c_^2^)/3
4705 reflections	(Δ/σ)_max_ = 0.001
271 parameters	Δρ_max_ = 0.33 e Å^−^^3^
0 restraints	Δρ_min_ = −0.27 e Å^−^^3^

### Special details

**Table:**

Geometry. All e.s.d.'s (except the e.s.d. in the dihedral angle between two l.s. planes) are estimated using the full covariance matrix. The cell e.s.d.'s are taken into account individually in the estimation of e.s.d.'s in distances, angles and torsion angles; correlations between e.s.d.'s in cell parameters are only used when they are defined by crystal symmetry. An approximate (isotropic) treatment of cell e.s.d.'s is used for estimating e.s.d.'s involving l.s. planes.
Refinement. Refinement of *F*^2^ against ALL reflections. The weighted *R*-factor *wR* and goodness of fit *S* are based on *F*^2^, conventional *R*-factors *R* are based on *F*, with *F* set to zero for negative *F*^2^. The threshold expression of *F*^2^ > σ(*F*^2^) is used only for calculating *R*-factors(gt) *etc*. and is not relevant to the choice of reflections for refinement. *R*-factors based on *F*^2^ are statistically about twice as large as those based on *F*, and *R*- factors based on ALL data will be even larger.

### Fractional atomic coordinates and isotropic or equivalent isotropic displacement parameters (Å^2^)

**Table:**

	*x*	*y*	*z*	*U*_iso_*/*U*_eq_	
C17	0.61732 (13)	0.9817 (2)	0.98635 (13)	0.0731 (5)	
H17A	0.6130	1.0629	1.0079	0.110*	
H17B	0.6397	0.9220	1.0258	0.110*	
H17C	0.5681	0.9524	0.9571	0.110*	
Zr1	0.5000	0.572850 (14)	0.7500	0.02355 (5)	
N1	0.48851 (5)	0.45657 (9)	0.63879 (6)	0.02849 (19)	
C5	0.53457 (7)	0.35957 (11)	0.64083 (7)	0.0320 (2)	
C1	0.43464 (7)	0.48587 (12)	0.57517 (7)	0.0322 (2)	
C2	0.42369 (9)	0.41586 (14)	0.51009 (8)	0.0426 (3)	
H2	0.3856	0.4369	0.4661	0.051*	
C4	0.52766 (9)	0.28548 (14)	0.57799 (8)	0.0449 (3)	
H4	0.5604	0.2182	0.5800	0.054*	
C3	0.47076 (10)	0.31419 (15)	0.51223 (9)	0.0502 (4)	
H3	0.4643	0.2650	0.4695	0.060*	
N3	0.68745 (7)	0.88262 (13)	0.91339 (7)	0.0443 (3)	
N4	0.74780 (9)	0.76062 (17)	0.84880 (10)	0.0600 (4)	
C12	0.73020 (8)	0.87419 (17)	0.86785 (9)	0.0468 (3)	
C16	0.66558 (9)	0.99384 (17)	0.93708 (10)	0.0539 (4)	
C13	0.75373 (9)	0.98893 (19)	0.84391 (10)	0.0580 (4)	
H13	0.7833	0.9879	0.8124	0.070*	
C15	0.68835 (12)	1.10440 (19)	0.91395 (13)	0.0694 (5)	
H15	0.6743	1.1819	0.9292	0.083*	
C14	0.73318 (11)	1.10031 (19)	0.86693 (12)	0.0675 (5)	
H14	0.7491	1.1759	0.8513	0.081*	
O1	0.40841 (5)	0.64343 (9)	0.65095 (5)	0.03362 (18)	
O2	0.34539 (6)	0.65095 (11)	0.52861 (6)	0.0505 (3)	
C6	0.39137 (7)	0.60152 (12)	0.58346 (7)	0.0333 (2)	
N2	0.5000	0.79516 (13)	0.7500	0.0288 (3)	
O5	0.42116 (5)	0.65285 (9)	0.80434 (5)	0.03414 (18)	
C8	0.45374 (7)	0.85771 (12)	0.77872 (7)	0.0326 (2)	
C11	0.40782 (7)	0.77095 (12)	0.81082 (7)	0.0332 (2)	
O6	0.36459 (7)	0.81504 (11)	0.84149 (7)	0.0520 (3)	
C9	0.45124 (9)	0.98904 (14)	0.77885 (10)	0.0483 (3)	
H9	0.4178	1.0321	0.7977	0.058*	
C10	0.5000	1.0542 (2)	0.7500	0.0603 (7)	
H10	0.5000	1.1425	0.7500	0.072*	
O4	0.64121 (6)	0.25978 (10)	0.72642 (6)	0.0482 (2)	
C7	0.59251 (7)	0.34202 (12)	0.71639 (7)	0.0324 (2)	
O3	0.58564 (5)	0.41882 (9)	0.76672 (5)	0.03380 (18)	
O7	0.74136 (9)	0.47442 (18)	0.85925 (12)	0.0865 (6)	
H3A	0.6742 (11)	0.810 (2)	0.9320 (12)	0.064 (6)*	
H4B	0.7294 (12)	0.690 (2)	0.8587 (12)	0.066 (6)*	
H4A	0.7747 (12)	0.755 (2)	0.8218 (12)	0.068 (6)*	
H7A	0.7728 (18)	0.425 (3)	0.8528 (17)	0.105 (10)*	
H7B	0.6993 (19)	0.445 (3)	0.8293 (19)	0.121 (12)*	

### Atomic displacement parameters (Å^2^)

**Table:**

	*U*^11^	*U*^22^	*U*^33^	*U*^12^	*U*^13^	*U*^23^
C17	0.0748 (13)	0.0714 (13)	0.0801 (14)	0.0028 (11)	0.0344 (11)	−0.0088 (11)
Zr1	0.02282 (7)	0.02414 (7)	0.02305 (8)	0.000	0.00640 (5)	0.000
N1	0.0303 (4)	0.0279 (4)	0.0274 (5)	−0.0010 (3)	0.0094 (4)	−0.0011 (4)
C5	0.0370 (6)	0.0285 (5)	0.0338 (6)	0.0001 (4)	0.0158 (5)	−0.0007 (5)
C1	0.0338 (5)	0.0335 (6)	0.0279 (6)	−0.0029 (5)	0.0080 (4)	−0.0008 (5)
C2	0.0499 (7)	0.0455 (7)	0.0289 (6)	−0.0053 (6)	0.0077 (5)	−0.0046 (6)
C4	0.0597 (8)	0.0364 (7)	0.0426 (7)	0.0051 (6)	0.0222 (7)	−0.0067 (6)
C3	0.0703 (10)	0.0455 (8)	0.0366 (7)	−0.0022 (7)	0.0196 (7)	−0.0127 (6)
N3	0.0399 (6)	0.0458 (6)	0.0432 (7)	−0.0095 (5)	0.0077 (5)	0.0018 (5)
N4	0.0627 (9)	0.0603 (9)	0.0665 (10)	−0.0172 (7)	0.0342 (8)	−0.0101 (8)
C12	0.0372 (6)	0.0571 (9)	0.0406 (7)	−0.0126 (6)	0.0048 (6)	0.0019 (7)
C16	0.0480 (8)	0.0519 (9)	0.0550 (9)	−0.0017 (7)	0.0069 (7)	−0.0012 (8)
C13	0.0472 (8)	0.0668 (11)	0.0538 (10)	−0.0144 (8)	0.0073 (7)	0.0165 (8)
C15	0.0661 (11)	0.0478 (9)	0.0845 (14)	0.0016 (8)	0.0102 (10)	0.0063 (9)
C14	0.0571 (10)	0.0577 (11)	0.0746 (13)	−0.0119 (8)	0.0026 (9)	0.0254 (9)
O1	0.0330 (4)	0.0357 (4)	0.0283 (4)	0.0059 (3)	0.0044 (3)	−0.0009 (3)
O2	0.0530 (6)	0.0550 (6)	0.0322 (5)	0.0155 (5)	−0.0024 (4)	0.0022 (5)
C6	0.0307 (5)	0.0356 (6)	0.0296 (6)	0.0004 (4)	0.0041 (4)	0.0018 (5)
N2	0.0306 (6)	0.0285 (6)	0.0260 (6)	0.000	0.0072 (5)	0.000
O5	0.0361 (4)	0.0330 (4)	0.0374 (5)	0.0024 (3)	0.0174 (4)	0.0019 (4)
C8	0.0353 (6)	0.0304 (6)	0.0308 (6)	0.0035 (5)	0.0089 (5)	−0.0012 (5)
C11	0.0331 (5)	0.0368 (6)	0.0306 (6)	0.0051 (5)	0.0112 (5)	0.0002 (5)
O6	0.0572 (6)	0.0494 (6)	0.0625 (7)	0.0113 (5)	0.0375 (6)	−0.0011 (5)
C9	0.0575 (8)	0.0322 (6)	0.0612 (10)	0.0056 (6)	0.0273 (8)	−0.0039 (6)
C10	0.0776 (16)	0.0267 (9)	0.0865 (19)	0.000	0.0399 (15)	0.000
O4	0.0482 (5)	0.0431 (5)	0.0556 (6)	0.0190 (4)	0.0197 (5)	0.0058 (5)
C7	0.0320 (5)	0.0292 (5)	0.0389 (6)	0.0022 (4)	0.0154 (5)	0.0037 (5)
O3	0.0314 (4)	0.0355 (4)	0.0324 (4)	0.0060 (3)	0.0072 (3)	0.0000 (4)
O7	0.0527 (8)	0.0816 (11)	0.1081 (13)	0.0094 (8)	0.0016 (8)	−0.0399 (10)

### Geometric parameters (Å, °)

**Table:**

C17—C16	1.491 (3)	N4—C12	1.320 (2)
C17—H17A	0.9600	N4—H4B	0.86 (2)
C17—H17B	0.9600	N4—H4A	0.82 (2)
C17—H17C	0.9600	C12—C13	1.409 (2)
Zr1—O5	2.2113 (9)	C16—C15	1.358 (3)
Zr1—O5^i^	2.2113 (9)	C13—C14	1.348 (3)
Zr1—O1^i^	2.2183 (11)	C13—H13	0.9300
Zr1—O1	2.2183 (11)	C15—C14	1.399 (3)
Zr1—O3^i^	2.2320 (9)	C15—H15	0.9300
Zr1—O3	2.2320 (9)	C14—H14	0.9300
Zr1—N2	2.3422 (15)	O1—C6	1.2828 (15)
Zr1—N1	2.3713 (10)	O2—C6	1.2280 (16)
Zr1—N1^i^	2.3713 (11)	N2—C8	1.3314 (14)
N1—C5	1.3297 (15)	N2—C8^i^	1.3314 (14)
N1—C1	1.3313 (16)	O5—C11	1.2824 (16)
C5—C4	1.3863 (18)	C8—C9	1.3845 (19)
C5—C7	1.4979 (19)	C8—C11	1.5062 (18)
C1—C2	1.3857 (18)	C11—O6	1.2246 (15)
C1—C6	1.4986 (18)	C9—C10	1.3834 (19)
C2—C3	1.379 (2)	C9—H9	0.9300
C2—H2	0.9300	C10—C9^i^	1.3834 (19)
C4—C3	1.383 (2)	C10—H10	0.9300
C4—H4	0.9300	O4—C7	1.2279 (15)
C3—H3	0.9300	C7—O3	1.2814 (15)
N3—C12	1.348 (2)	O7—H7A	0.82 (3)
N3—C16	1.362 (2)	O7—H7B	0.87 (4)
N3—H3A	0.91 (2)		
			
C16—C17—H17A	109.5	C1—C2—H2	120.9
C16—C17—H17B	109.5	C3—C4—C5	118.26 (13)
H17A—C17—H17B	109.5	C3—C4—H4	120.9
C16—C17—H17C	109.5	C5—C4—H4	120.9
H17A—C17—H17C	109.5	C2—C3—C4	119.92 (13)
H17B—C17—H17C	109.5	C2—C3—H3	120.0
O5—Zr1—O5^i^	135.19 (5)	C4—C3—H3	120.0
O5—Zr1—O1^i^	86.35 (4)	C12—N3—C16	124.41 (15)
O5^i^—Zr1—O1^i^	78.94 (4)	C12—N3—H3A	118.6 (13)
O5—Zr1—O1	78.94 (4)	C16—N3—H3A	116.9 (13)
O5^i^—Zr1—O1	86.35 (4)	C12—N4—H4B	124.6 (14)
O1^i^—Zr1—O1	140.83 (5)	C12—N4—H4A	118.8 (16)
O5—Zr1—O3^i^	77.70 (4)	H4B—N4—H4A	116 (2)
O5^i^—Zr1—O3^i^	140.11 (3)	N4—C12—N3	118.80 (15)
O1^i^—Zr1—O3^i^	133.65 (3)	N4—C12—C13	124.11 (16)
O1—Zr1—O3^i^	78.30 (4)	N3—C12—C13	117.09 (17)
O5—Zr1—O3	140.11 (3)	C15—C16—N3	118.41 (18)
O5^i^—Zr1—O3	77.70 (4)	C15—C16—C17	125.88 (19)
O1^i^—Zr1—O3	78.30 (4)	N3—C16—C17	115.71 (16)
O1—Zr1—O3	133.65 (3)	C14—C13—C12	119.64 (17)
O3^i^—Zr1—O3	86.71 (5)	C14—C13—H13	120.2
O5—Zr1—N2	67.59 (2)	C12—C13—H13	120.2
O5^i^—Zr1—N2	67.59 (2)	C16—C15—C14	119.2 (2)
O1^i^—Zr1—N2	70.41 (3)	C16—C15—H15	120.4
O1—Zr1—N2	70.41 (3)	C14—C15—H15	120.4
O3^i^—Zr1—N2	136.64 (2)	C13—C14—C15	121.26 (17)
O3—Zr1—N2	136.64 (2)	C13—C14—H14	119.4
O5—Zr1—N1	135.80 (4)	C15—C14—H14	119.4
O5^i^—Zr1—N1	71.15 (4)	C6—O1—Zr1	126.56 (8)
O1^i^—Zr1—N1	137.80 (3)	O2—C6—O1	124.76 (12)
O1—Zr1—N1	66.77 (4)	O2—C6—C1	121.01 (12)
O3^i^—Zr1—N1	68.96 (4)	O1—C6—C1	114.21 (11)
O3—Zr1—N1	66.91 (4)	C8—N2—C8^i^	120.65 (15)
N2—Zr1—N1	121.11 (2)	C8—N2—Zr1	119.67 (8)
O5—Zr1—N1^i^	71.15 (4)	C8^i^—N2—Zr1	119.67 (8)
O5^i^—Zr1—N1^i^	135.80 (4)	C11—O5—Zr1	126.32 (8)
O1^i^—Zr1—N1^i^	66.77 (4)	N2—C8—C9	121.48 (12)
O1—Zr1—N1^i^	137.80 (3)	N2—C8—C11	112.92 (11)
O3^i^—Zr1—N1^i^	66.91 (4)	C9—C8—C11	125.59 (11)
O3—Zr1—N1^i^	68.96 (4)	O6—C11—O5	126.19 (12)
N2—Zr1—N1^i^	121.11 (3)	O6—C11—C8	120.34 (12)
N1—Zr1—N1^i^	117.79 (5)	O5—C11—C8	113.43 (10)
C5—N1—C1	120.19 (11)	C10—C9—C8	117.95 (14)
C5—N1—Zr1	119.72 (8)	C10—C9—H9	121.0
C1—N1—Zr1	120.07 (8)	C8—C9—H9	121.0
N1—C5—C4	121.64 (13)	C9—C10—C9^i^	120.45 (19)
N1—C5—C7	112.91 (10)	C9—C10—H10	119.8
C4—C5—C7	125.46 (12)	C9^i^—C10—H10	119.8
N1—C1—C2	121.68 (12)	O4—C7—O3	125.20 (13)
N1—C1—C6	112.04 (10)	O4—C7—C5	120.70 (12)
C2—C1—C6	126.27 (12)	O3—C7—C5	114.10 (10)
C3—C2—C1	118.30 (14)	C7—O3—Zr1	126.28 (8)
C3—C2—H2	120.9	H7A—O7—H7B	103 (3)
			
O5—Zr1—N1—C5	−137.47 (8)	C2—C1—C6—O1	174.88 (12)
O5^i^—Zr1—N1—C5	86.13 (9)	O5—Zr1—N2—C8	−2.02 (7)
O1^i^—Zr1—N1—C5	38.83 (11)	O5^i^—Zr1—N2—C8	177.98 (7)
O1—Zr1—N1—C5	−179.67 (9)	O1^i^—Zr1—N2—C8	−96.24 (7)
O3^i^—Zr1—N1—C5	−93.76 (9)	O1—Zr1—N2—C8	83.76 (7)
O3—Zr1—N1—C5	1.82 (8)	O3^i^—Zr1—N2—C8	37.42 (7)
N2—Zr1—N1—C5	133.57 (8)	O3—Zr1—N2—C8	−142.58 (7)
N1^i^—Zr1—N1—C5	−46.43 (8)	N1—Zr1—N2—C8	129.04 (7)
O5—Zr1—N1—C1	40.43 (11)	N1^i^—Zr1—N2—C8	−50.96 (7)
O5^i^—Zr1—N1—C1	−95.97 (9)	O5—Zr1—N2—C8^i^	177.98 (7)
O1^i^—Zr1—N1—C1	−143.27 (8)	O5^i^—Zr1—N2—C8^i^	−2.02 (7)
O1—Zr1—N1—C1	−1.77 (8)	O1^i^—Zr1—N2—C8^i^	83.76 (7)
O3^i^—Zr1—N1—C1	84.14 (9)	O1—Zr1—N2—C8^i^	−96.24 (7)
O3—Zr1—N1—C1	179.72 (10)	O3^i^—Zr1—N2—C8^i^	−142.58 (7)
N2—Zr1—N1—C1	−48.53 (9)	O3—Zr1—N2—C8^i^	37.42 (7)
N1^i^—Zr1—N1—C1	131.47 (9)	N1—Zr1—N2—C8^i^	−50.96 (7)
C1—N1—C5—C4	−0.84 (18)	N1^i^—Zr1—N2—C8^i^	129.04 (7)
Zr1—N1—C5—C4	177.05 (10)	O5^i^—Zr1—O5—C11	1.94 (9)
C1—N1—C5—C7	178.88 (10)	O1^i^—Zr1—O5—C11	72.24 (10)
Zr1—N1—C5—C7	−3.22 (13)	O1—Zr1—O5—C11	−71.27 (10)
C5—N1—C1—C2	1.35 (18)	O3^i^—Zr1—O5—C11	−151.55 (11)
Zr1—N1—C1—C2	−176.54 (10)	O3—Zr1—O5—C11	139.09 (10)
C5—N1—C1—C6	−177.21 (10)	N2—Zr1—O5—C11	1.94 (9)
Zr1—N1—C1—C6	4.90 (13)	N1—Zr1—O5—C11	−110.25 (10)
N1—C1—C2—C3	−0.5 (2)	N1^i^—Zr1—O5—C11	138.93 (11)
C6—C1—C2—C3	177.80 (13)	C8^i^—N2—C8—C9	0.92 (11)
N1—C5—C4—C3	−0.4 (2)	Zr1—N2—C8—C9	−179.08 (11)
C7—C5—C4—C3	179.87 (13)	C8^i^—N2—C8—C11	−178.02 (11)
C1—C2—C3—C4	−0.8 (2)	Zr1—N2—C8—C11	1.98 (11)
C5—C4—C3—C2	1.2 (2)	Zr1—O5—C11—O6	−179.35 (11)
C16—N3—C12—N4	179.94 (16)	Zr1—O5—C11—C8	−1.59 (15)
C16—N3—C12—C13	0.3 (2)	N2—C8—C11—O6	177.52 (11)
C12—N3—C16—C15	−0.3 (2)	C9—C8—C11—O6	−1.4 (2)
C12—N3—C16—C17	179.07 (16)	N2—C8—C11—O5	−0.38 (15)
N4—C12—C13—C14	−179.53 (18)	C9—C8—C11—O5	−179.28 (14)
N3—C12—C13—C14	0.1 (2)	N2—C8—C9—C10	−1.8 (2)
N3—C16—C15—C14	0.0 (3)	C11—C8—C9—C10	177.02 (11)
C17—C16—C15—C14	−179.37 (19)	C8—C9—C10—C9^i^	0.86 (10)
C12—C13—C14—C15	−0.4 (3)	N1—C5—C7—O4	−176.83 (11)
C16—C15—C14—C13	0.4 (3)	C4—C5—C7—O4	2.9 (2)
O5—Zr1—O1—C6	−154.09 (10)	N1—C5—C7—O3	3.20 (15)
O5^i^—Zr1—O1—C6	68.45 (10)	C4—C5—C7—O3	−177.09 (12)
O1^i^—Zr1—O1—C6	135.95 (10)	O4—C7—O3—Zr1	178.19 (10)
O3^i^—Zr1—O1—C6	−74.53 (10)	C5—C7—O3—Zr1	−1.84 (14)
O3—Zr1—O1—C6	−0.71 (12)	O5—Zr1—O3—C7	135.01 (9)
N2—Zr1—O1—C6	135.95 (10)	O5^i^—Zr1—O3—C7	−74.37 (10)
N1—Zr1—O1—C6	−2.60 (9)	O1^i^—Zr1—O3—C7	−155.43 (10)
N1^i^—Zr1—O1—C6	−108.97 (10)	O1—Zr1—O3—C7	−1.71 (12)
Zr1—O1—C6—O2	−172.23 (10)	O3^i^—Zr1—O3—C7	68.68 (9)
Zr1—O1—C6—C1	5.94 (15)	N2—Zr1—O3—C7	−111.32 (9)
N1—C1—C6—O2	171.61 (12)	N1—Zr1—O3—C7	0.18 (9)
C2—C1—C6—O2	−6.9 (2)	N1^i^—Zr1—O3—C7	135.18 (10)
N1—C1—C6—O1	−6.64 (15)		

Symmetry codes: (i) −*x*+1, *y*, −*z*+3/2.

### Hydrogen-bond geometry (Å, °)

**Table:**

*D*—H···*A*	*D*—H	H···*A*	*D*···*A*	*D*—H···*A*
O7—H7B···O3	0.87 (4)	2.09 (4)	2.938 (2)	164 (3)
O7—H7A···O6^ii^	0.82 (3)	2.14 (3)	2.9568 (19)	173 (3)
N4—H4B···O1^i^	0.86 (2)	2.57 (2)	3.1755 (18)	127.6 (17)
N4—H4B···O7	0.86 (2)	2.28 (2)	3.027 (3)	144.1 (18)
N4—H4A···O4^iii^	0.82 (2)	2.05 (2)	2.861 (2)	168 (2)
N3—H3A···O2^i^	0.91 (2)	1.91 (2)	2.8194 (18)	175.1 (18)

Symmetry codes: (ii) *x*+1/2, *y*−1/2, *z*; (i) −*x*+1, *y*, −*z*+3/2; (iii) −*x*+3/2, *y*+1/2, −*z*+3/2.
